# Decursin and decursinol angelate inhibit estrogen-stimulated and estrogen-independent growth and survival of breast cancer cells

**DOI:** 10.1186/bcr1790

**Published:** 2007-11-06

**Authors:** Cheng Jiang, Junming Guo, Zhe Wang, Bingxiu Xiao, Hyo-Jung Lee, Eun-Ok Lee, Sung-Hoon Kim, Junxuan Lu

**Affiliations:** 1 The Hormel Institute, University of Minnesota, 801 16th Avenue NE, Austin, MN 55912, USA; 2 Ningbo University School of Medicine, Ningbo, Zhejiang Province, China; 3 Cancer Preventive Material Development Research Center (CPMDRC), College of Oriental Medicine, Kyunghee University, Hoegi-dong, Dongdaemun-gu, Seoul 131-701, Republic of Korea

## Abstract

**Introduction:**

Estrogen and estrogen receptor (ER)-mediated signaling are crucial for the etiology and progression of human breast cancer. Attenuating ER activities by natural products is a promising strategy to decrease breast cancer risk. We recently discovered that the pyranocoumarin compound decursin and its isomer decursinol angelate (DA) have potent novel antiandrogen receptor signaling activities. Because the ER and the androgen receptor belong to the steroid receptor superfamily, we examined whether these compounds affected ER expression and signaling in breast cancer cells.

**Methods:**

We treated estrogen-dependent MCF-7 and estrogen-independent MDA MB-231 human breast cancer cells with decursin and DA, and examined cell growth, apoptosis, and ERα and ERβ expression in both cell lines – and, in particular, estrogen-stimulated signaling in the MCF-7 cells. We compared these compounds with decursinol to determine their structure-activity relationship.

**Results:**

Decursin and DA exerted growth inhibitory effects on MCF-7 cells through G_1 _arrest and caspase-mediated apoptosis. These compounds decreased ERα in MCF-7 cells at both mRNA and protein levels, and suppressed estrogen-stimulated genes. Decursin and the pure antiestrogen Faslodex™ exerted an additive growth inhibitory effect on MCF-7 cells. In MDA MB-231 cells, these compounds induced cell-cycle arrests in the G_1 _and G_2 _phases as well as inducing apoptosis, accompanied by an increased expression of ERβ. In contrast, decursinol, which lacks the side chain of decursin and DA, did not have these cellular and molecular activities at comparable concentrations.

**Conclusion:**

The side chain of decursin and DA is crucial for their anti-ER signaling and breast cancer growth inhibitory activities. These data provide mechanistic rationales for validating the chemopreventive and therapeutic efficacy of decursin and its derivatives in preclinical animal models of breast cancer.

## Introduction

Breast cancer is the most commonly diagnosed nonskin malignancy among American women, accounting for approximately 32% (211,000 cases) of all new cancer cases per year [1]. It is the second leading cause of cancer death in US women, claiming the lives of 41,000 annually. The female hormone estrogen and its classic intracellular receptor – estrogen receptor (ER) alpha – are crucial for breast development and are also causally linked to the etiology and progression of breast cancer and gynecologic cancers [2-4]. The genomic effects of estrogen are principally mediated by ERα [3], the activity of which is counter-balanced by the inhibitory ERβ [3,5-8].

Estrogen, through its ERα-mediated genomic effects and nongenomic effects, induces breast epithelial cell proliferation and suppresses apoptosis [3,9]. Since estrogen activity can be modulated by agonists as well as by antagonists, compounds that interfere with ERα function have proved useful in the treatment and chemoprevention of ER-positive breast cancer [10]. In fact, the classic nonsteroidal antiestrogen tamoxifen is the first Food and Drug Administration-approved chemopreventive agent for breast cancer. Because of its partial ER agonist activity, however, tamoxifen causes some adverse effects, including an increase of the risk of uterine endometrial cancer in women after prolonged use [11]; therefore women are not allowed to use this drug for longer than 5 years. Novel agents that target ERα signaling as well as estrogen production without agonist activities will be desirable for breast cancer chemoprevention and treatment, and for women who have finished the prescribed course of tamoxifen [12-15].

The root of Korean medicinal herb *Angelica gigas *Nakai (also known as Cham Dang Gui) has been used in traditional Oriental herbal medicine for thousands of years for treating female afflictions and is regarded by herbalists as 'female Ginseng' for its hemopoietic and health-promoting activities [16]. These known medicinal properties, however, were based on using boiling water to extract the active ingredients or chemicals. An often underappreciated principle of herbal medicine is that changing the extraction solvents can lead to the recovery of different chemicals and therefore to novel medicinal and pharmacological properties. In fact, many pyranocoumarin compounds have been identified from the alcohol extract of *A. gigas *Nakai [17,18] with diverse pharmacological activities including cytotoxic action against leukemia cells [19-22], antibacterial action [23], pain-killing action [24] and antimemory loss action [25].

**Figure 1:**
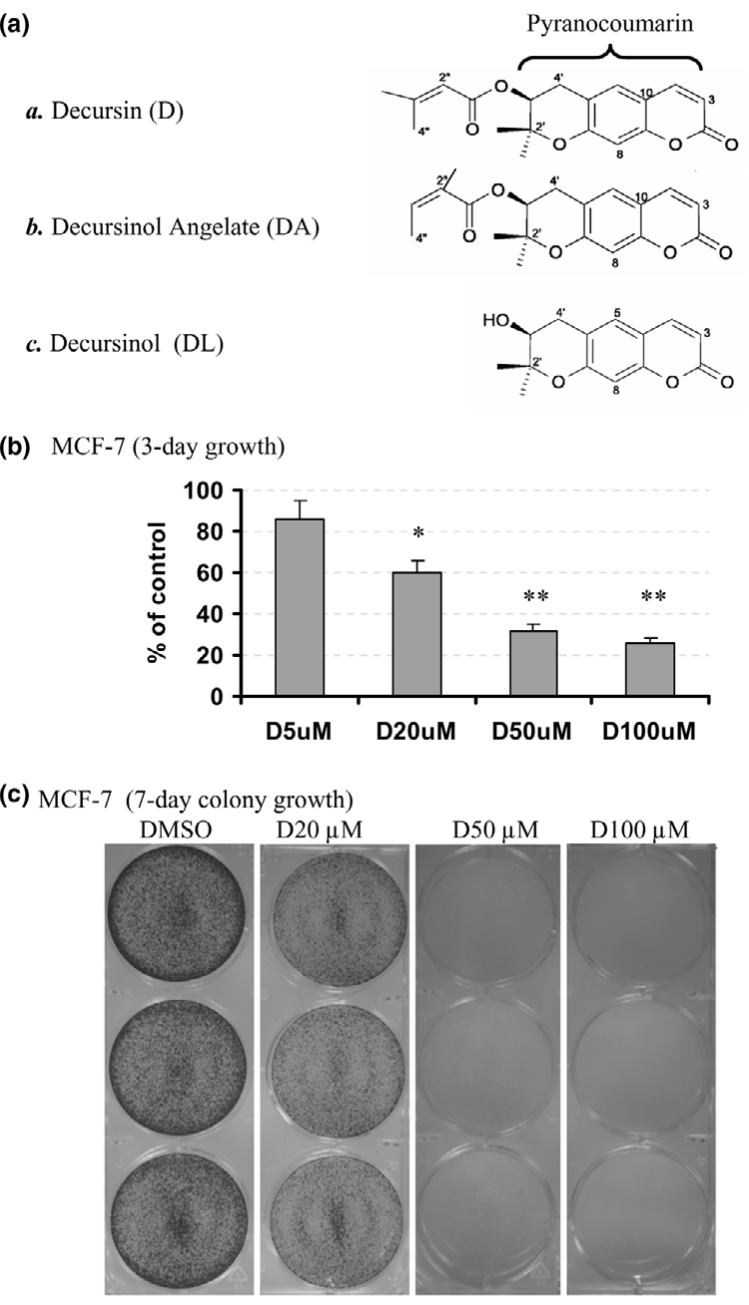
Growth inhibition of MCF-7 cells by decursin. **(a) **Chemical structure of pyranocoumarin compounds isolated from *Angelica gigas *root. **(b) **Growth inhibitory effects of decursin on exponentially growing MCF-7 cells (30–40% confluent) in T25 flasks. Exposure to different decursin concentrations (D5 μM, D20 μM, D50 μM, D100 μM) was in complete medium for 3 days. Results based on two separate experiments (mean ± standard deviation). *P < 0.05, **P < 0.001 versus control. **(c) **Inhibition of long-term colony growth of MCF-7 cells. Sparsely seeded cells (~10% confluent) in six-well plates were exposed to different decursin concentrations (D20 μM, D50 μM, D100 μM) in the complete medium for 7 days. After fixation with 1% glutaraldehyde, the cells remaining attached were stained *in situ *with 0.02% crystal violet and were photographed. DMSO, dimethylsulfoxide.

We recently discovered that the pyranocoumarin compound decursin and its isomer decursinol angelate (DA) (Figure 1) from the ethanol extract of dried *A. gigas *Nakai root have potent and novel antiandrogen receptor (anti-AR) signaling activities *in vitro *[26,27]. They inhibit the androgen-stimulated translocation of the AR from the cytosol to the nucleus, decrease the AR protein level in part through increased proteasomal degradation, and suppress AR transactivation activity [26,27]. In addition, these compounds have been shown to inhibit the growth of human leukemia cell lines and androgen-independent prostate cancer cell lines [28], and to increase the survival of mice carrying inoculated sarcoma cells *in vivo *[29]. Because the ER and the AR belong to the steroid receptor superfamily, we sought to determine whether these pyranocoumarin compounds exerted similar activities against ER expression and function in breast cancer cells.

In the present article we report that decursin and DA potently inhibit estrogen-stimulated mitogenesis at micromolar concentrations in the estrogen-dependent MCF-7 human breast cancer cells through G_1 _arrest and caspase-mediated apoptosis. The effects are associated with downregulating ERα protein abundance and transactivation functions. In addition, decursin and the steroidal pure antiestrogen Faslodex™ (ICI 182,780 or fulvestrant) [15,30] exert additive growth inhibitory activity in MCF-7 cells. Furthermore, these compounds induce cell-cycle arrest at the G_1 _or G_2 _phase and induce apoptosis in the estrogen-independent MDA231 human breast cancer cells, accompanied by an upregulation of ERβ. We report also the response patterns of selected cell-cycle and mitogenic signaling molecules in these two cell lines.

## Materials and methods

### Natural product chemicals

Decursin, DA and decursinol were extracted and purified as described in previous publications [26,27].

### Cell culture and treatments

MCF-7 cells (expressing high levels of ERα) and MDA MB-231 cells (ERα-negative, expressing ERβ) were obtained from the American Type Culture Collection (Manassas, VA, USA). The MCF-7 cells were grown in Eagle's minimal essential medium (American Type Culture Collection) supplemented with 10 μg/ml insulin and 10% FBS (Atlanta Biologicals, Lawrenceville, GA, USA) in a humidified incubator with 5% CO_2_. The MDA MB-231 cells were grown in Leibovitz's L-15 medium (American Type Culture Collection) supplemented with 10% FBS (Atlanta Biologicals) in a humidified incubator with atmospheric CO_2_. When cells were ~50% confluent (usually 2 days after plating for MCF-7 cells and 3 days after plating for MDA MB-231 cells), the medium was changed and treatment with compounds was started in fresh medium.

In experiments where estrogen stimulation was required, the spent medium was removed and the adherent MCF-7 cells were rinsed gently with phenol-red free improved minimal essential medium (PRF-IMEM) without serum and insulin (Biosource, Rockville, MD, USA) to remove leftover estrogen. PRF-IMEM with 5% charcoal-stripped FBS (Atlanta Biologicals) without insulin was then added for estrogen deprivation. Estrogen stimulation was provided by 17β-estradiol (Sigma Chemical Company, St Louis, MO, USA). Faslodex™ (ICI 182,780; Tocris Bioscience, Avonmouth, UK) and/or an active metabolite of tamoxifen, 4-hydroxytamoxifen (4-HT) (LKT Labs, St Paul, MN, USA), were used as positive antiestrogen agents for comparison with decursin and DA in key experiments.

### Cell growth and cell death (apoptosis) assays

The effects of compounds on breast cancer cell numbers were estimated either by counting cells with a hemacytometer or by crystal violet staining of adherent cells. Cell-cycle analyses were carried out with propidium iodide staining according to Krishan's protocol [31] and flow cytometry using a FACSCaliber flow cytometer (Becton-Dickinson, San Jose, CA, USA). Cell death was detected either by an ELISA kit for nucleosomal DNA fragmentation with an antibody sandwich assay that detects DNA and associated histones (Roche Diagnostics Corporation, Indianapolis, IN, USA) or by an immunoblot analysis of the caspase-mediated cleavage of poly(ADP-ribose) polymerase (PARP) as described previously [26].

### Reverse transcriptase-polymerase chain reaction

Estrogen-deprived MCF-7 cells were given fresh media containing decursin, DA, decursinol, Faslodex™ and 1 nM 17β-estradiol. After exposure for 24 hours, the attached cells underwent RNA extraction using an RNeasy kit (catalogue number 74104) purchased from Qiagen (Valencia, CA, USA). The mRNA level was estimated by RT-PCR in the linear range of detection as follows. Reverse transcription was performed with 4 μg total RNA using Oligo(dT) primers according to a SuperScript™ II RT kit (catalogue number 18064-014; GIBCO-BRL, Foster City, CA, USA). PCR amplification was performed using the HotStarTaq Master Mix Kit (catalogue number 203445; Qiagen). The PCR conditions were 95°C for 15 minutes for denaturation and then, depending on the different primers, a variable number of cycles of 95°C for 1 minute, 63°C for 1 minute and 72°C for 1 minute, and a final extension at 72°C for 10 minutes. The PCR products were fractionated on 1.5% agarose gels containing ethidium bromide. The gels were visualized under UV illumination and photographed by a digital image capture system (KODAK 1D3.6; Digital Science, Rochester, NY, USA).

Oligonucleotide primers were synthesized by Sigma-Genosys (The Woodlands, TX, USA) as follows: *pS2 *gene (220 bp, 22 cycles), forward 5'-TTT GGA GCA GAG AGG AGG CAA TGG-3' and reverse 5'-TGG TAT TAG GAT AGA AGC ACC AGG G-3' ; *ERα *gene (650 bp, 35 cycles), forward 5'-TAC TGC ATC AGA TCC AAG GG-3' and reverse 5'-ATC AAT GGT GCA CTG GTT GG-3' ; and housekeeping gene glyceraldehyde-3-phosphate dehydrogenase (230 bp, 25 cycles), forward 5'-TCA AGA AGG TGG TGA AGC AG-3' and reverse 5'-CTT ACT CCT TGG AGG CCA TG-3'.

### Immunoblotting

Cell lysate was prepared in radioimmunoprecipitation assay buffer as described previously [26]. Supernatants after centrifugation (14,000 × *g*, 20 min, 4°C) were recovered and the protein content was quantified by the Lowry assay. Proteins were size-separated by electrophoresis on SDS-PAGE gels and were electroblotted onto nitrocellulose membranes. Immunodetection was carried out with antibodies for ERα, ERβ, and cyclin D_1 _purchased from Santa Cruz Biotechnology Inc. (Santa Cruz, CA, USA) using enhanced chemofluorescence detection with a Storm machine (Molecular Dynamics, Sunnyvale, CA, USA). Cleaved PARP as a marker of caspase-mediated apoptosis, phospho-ERK1/2, total ERK1/2, P21Cip1 and P27Kip1 were immunoblotted with antibodies from Cell Signaling Technologies (Beverly, MA, USA). β-Actin was detected as a protein loading control.

### Estrogen receptor alpha nuclear/cytosol distribution

Subconfluent MCF-7 cells were incubated with PRF-IMEM without serum and insulin for 48 hours to decrease background mitogen signaling. Fresh medium without or with 1 nM 17β-estradiol was added. Cells were harvested at different time points. Nuclear and cytosolic fractions were prepared using NE-PER Nuclear and Cytoplasmic Extraction Reagents kit (Pierce, Rockford, IL, USA) for western blot analyses. PARP and α-tubulin were detected as markers of nuclear and cytosol proteins, respectively. For testing the effect of decursin, estrogen-deprived MCF-7 cells were pre-treated with decursin for 2 hours and were stimulated with 1 nM 17β-estradiol for 1 hour (total decursin exposure time of 3 hours). The cells were subjected to fractionation to yield nuclear and cytosolic fractions for western blot analyses. Separate sets of flasks were treated identically in parallel and used for total cell lysate preparation for western blot analyses of the AR protein level.

### Estrogen-response element transactivation

To determine the effects of decursin versus decursinol on the transactivation of estrogen-response element (ERE) activity in MCF-7 cells, we performed a transient transfection assay with an ERE-luciferase report plasmid (a generous gift from Dr Allen Gao, Roswell Park Cancer Institute, Buffalo, NY, USA) [32]. An aliquot of 1 × 10^5 ^MCF-7 cells was placed in a 12-well plate and co-transfected with 0.5 μg ERE-luciferase plasmid and 1.0 μg pSV-β-galactosidase reporter vector using jetPEI cationic polymer transfection reagent (Polyplus-Transfection Inc. New York, NY, USA) in PRF-IMEM medium without serum and insulin. After transfection for 24 hours, the cells were treated with different concentrations of decursin, decursinol or dimethylsulfoxide vehicle in the absence or presence of 1 nM 17β-estradiol. Cells extracts were prepared for luciferase activity and β-galactosidase activity, and the luciferase data were normalized to β-galactosidase.

### Statistical evaluations

When appropriate and necessary, analyses of variance and post-hoc comparisons (Bonferroni multiple comparison test) were used to evaluate statistical significance.

## Results

### Growth inhibition of MCF-7 cells by decursin

To assess the effect of decursin on MCF-7 cell growth, we first evaluated the cell number after 72 hours of treatment in complete medium containing 10% FBS (T25 flasks, triplicate). As shown in Figure 1b, decursin decreased the MCF-7 cell number in a concentration-dependent manner (*P *< 0.01 for 20 μM, *P *< 0.001 for 50 μM and 100 μM). In a colony growth assay of sparsely seeded cells, we detected cell colonies by staining with diluted crystal violet after fixation (Figure 1c). The number and size of crystal violet stainable colonies on day 7 of treatment were substantially decreased by a single exposure at 20 μM and higher concentrations (Figure 1c).

### Requirement of side chain for cell-cycle arrest and apoptosis induction in estrogen-dependent and estrogen-independent breast cancer cells

**Figure 2:**
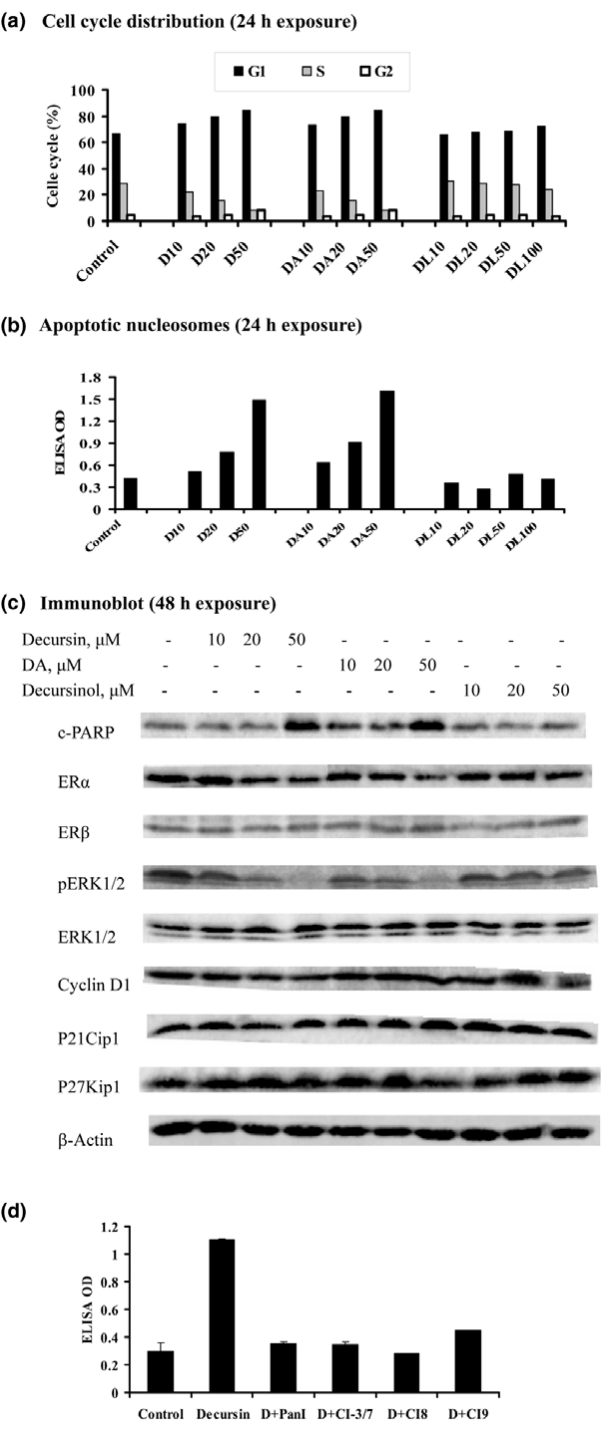
Apoptotic and cell-cycle effects of pyranocoumarins in MCF-7 cells. **(a) **Cell-cycle distribution: cells were treated with decursin (D), decursinol angelate (DA) or decursinol (DL) at different exposure concentrations (10 μM, 20 μM, 50 μM) for 24 hours in complete medium. Bar height represents average of duplicate flasks, variation less than 2%. **(b) **Apoptotic DNA nucleosomes detected by the Death ELISA kit in MCF-7 cells after 24 hours of treatment in complete medium. Bar height represents average of duplicate flasks, variation less than 2%. **(c) **Western blot analysis detection of cleaved poly(ADP-ribose) polymerase (c-PARP), estrogen receptors (ERs) and selected mitogen signaling and cell-cycle regulator proteins after 48 hours of treatment in complete medium. **(d) **Inhibition of decursin (50 μM)-induced apoptosis by 10 μM caspase inhibitors (CIs). PanI, Pan inhibitor (Q-VD-OPH); CI-3/7, zDEVDfmk; CI8, zIETDfmk; and CI9, zLEHDfmk (Enzyme Systems Products, Dublin, CA, USA). Inhibitors were added 2 hours prior to decursin treatment for an additional 24 hours. Mean ± standard deviation, triplicate determinations.

**Figure 3:**
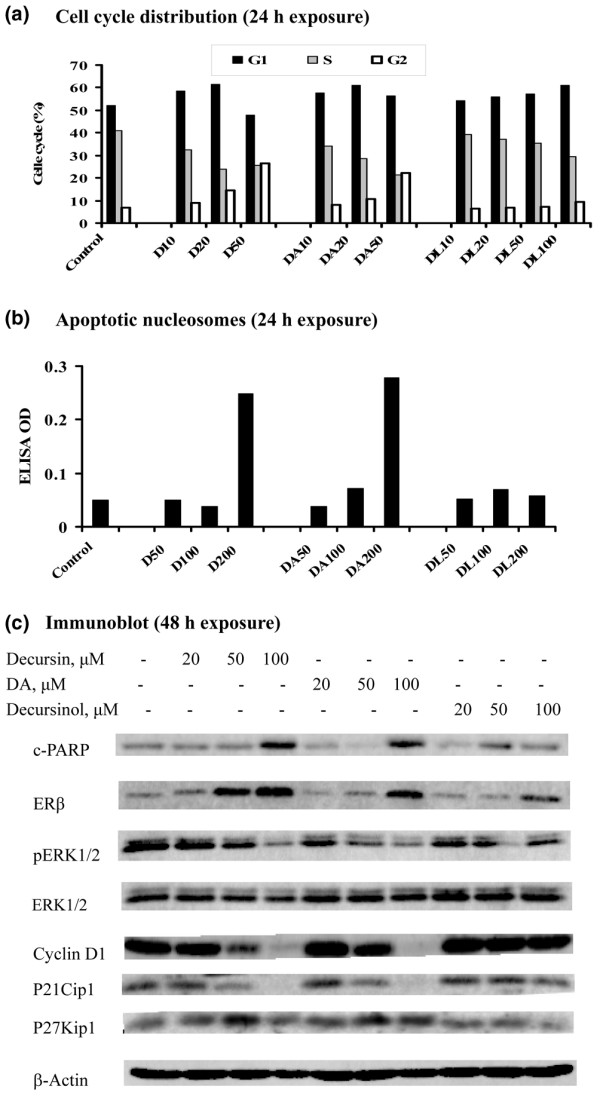
Apoptotic and cell-cycle effects of pyranocoumarins on MDA-MB231 estrogen-independent breast cancer cells. **(a) **Cell-cycle distribution after 24 hours of treatment with decursin (D), decursinol angelate (DA) or decursinol (DL) at different exposure concentrations (10 μM, 20 μM, 50 μM) in complete medium. Cells prepared as in Figure 2 for flow cytometric analysis. Bar height represents average of duplicate flasks, variation less than 2%. **(b) **Apoptosis detected by the DNA fragmentation Death ELISA kit in MDA-MB231 cells after 24 hours of treatment with different exposure concentrations (50 μM, 100 μM, 200 μM) in complete medium. **(c) **Western blot analysis detection of cleaved poly(ADP-ribose) polymerase (c-PARP), estrogen receptor (ER)β and selected mitogen signaling and cell-cycle regulator proteins after 48 hours of treatment in complete medium.

To examine whether an induction of cell-cycle arrest and apoptosis contributed to the inhibition of breast cancer cell growth by decursin, and to determine the chemical structure requirement for such activities, we evaluated the effects of decursin and of its structural isomer DA versus decursinol in complete medium on the cell-cycle distribution and apoptosis in MCF-7 cells (Figure 2) and in MDA-MB231 estrogen-independent breast cancer cells (Figure 3).

When analyzed by flow cytometry at 24 hours of treatment (Figure 2a), decursin and DA increased the G_1_-phase population of MCF-7 cells and decreased the S-phase cells (G_1 _arrest) in the range of 10–50 μM in a concentration-dependent manner (Figure 2a). ELISA detection of the apoptotic DNA fragmentation at 24 hours (Figure 2b) as well as western blot analysis for cleaved PARP at 48 hours of treatment (Figure 2c) indicated increased caspase-mediated apoptosis at 50 μM decursin or 50 μM DA in MCF-7 cells, in spite of a lack of caspase-3 in these cells [33]. The cell-cycle arrest and apoptosis effects were accompanied by decreased ERα abundance and decreased ERK1/2 phosphorylation without changes of ERβ, cyclin D_1 _and the two cyclin-dependent kinase inhibitor proteins P21Cip1 and P27Kip1 (Figure 2c). The apoptosis could be blocked by the pan-caspase inhibitor Q-VD-OPH, or could be inhibited by the specific inhibitors of caspase 3/caspase 7, caspase 8, and caspase 9 in MCF-7 cells (Figure 2d). Decursinol at the tested concentrations, however, did not cause cell-cycle arrest (Figure 2a) or cell death (Figure 2b,c) and caused little or minimal changes in ERα abundance and ERK1/2 phosphorylation (Figure 2c).

For the estrogen-independent MDA MB-231 cells, decursin and DA induced G_1 _arrest in the range 10–20 μM but induced G_2 _arrest at 50 μM (Figure 3a). The G_2 _arrest effect was associated with an increased abundance of the growth-inhibitory ERβ protein and increased expression of P27Kip1 (Figure 3c). This was consistent with the reported G_2_-arresting activity of ERβ [34] and with P27Kip1 as a mediator of cell-cycle arrest action of ERβ [35]. Decursinol, however, did not alter the cell-cycle distribution pattern – except at 100 μM, a concentration that increased G_1_-arrested cells (Figure 3a). Apoptosis was induced in MDA MB-231 cells by decursin and DA, but required at least twice as much of these compounds as in MCF-7 cells (Figure 3b,c). The apoptotic events were associated not only with increased abundance of ERβ protein, but also with a decreased phosphorylation of ERK1/2 and decreased cyclin D_1_, and a paradoxical decrease of P21Cip1. The caspase inhibitors blocked decursin-induced apoptosis in MDA MB231 cells in the same fashion as in MCF-7 cells (data not shown). Decursinol did not induce apoptosis at comparable concentrations and caused a modest increase of ERβ protein and a decrease of pERK1/2 and P21Cip1 at an exposure concentration of 100 μM, which induced G_1 _arrest.

Taken together, these results showed that the side chain on decursin or DA was critical for the induction of cell-cycle arrests and apoptosis in both breast cancer cell lines, regardless of their estrogen-dependence status. Moreover, the estrogen-dependent MCF-7 cells were more sensitive than the estrogen-independent MDA-MB231 cells to the actions of these effective compounds. The growth inhibitory effects involved common pathways such as the inactivation of the mitogen signaling ERK1/2, as well as distinct targets such as ERα in MCF-7 cells, and ERβ and P27Kip1 in MDA-MB231 cells. In both cell lines, we did not detect an upregulation of P21Cip1 protein in spite of cell-cycle arrests.

### Effects of decursin or decursinol angelate on estrogen-stimulated growth of MCF-7 cells

**Figure 4:**
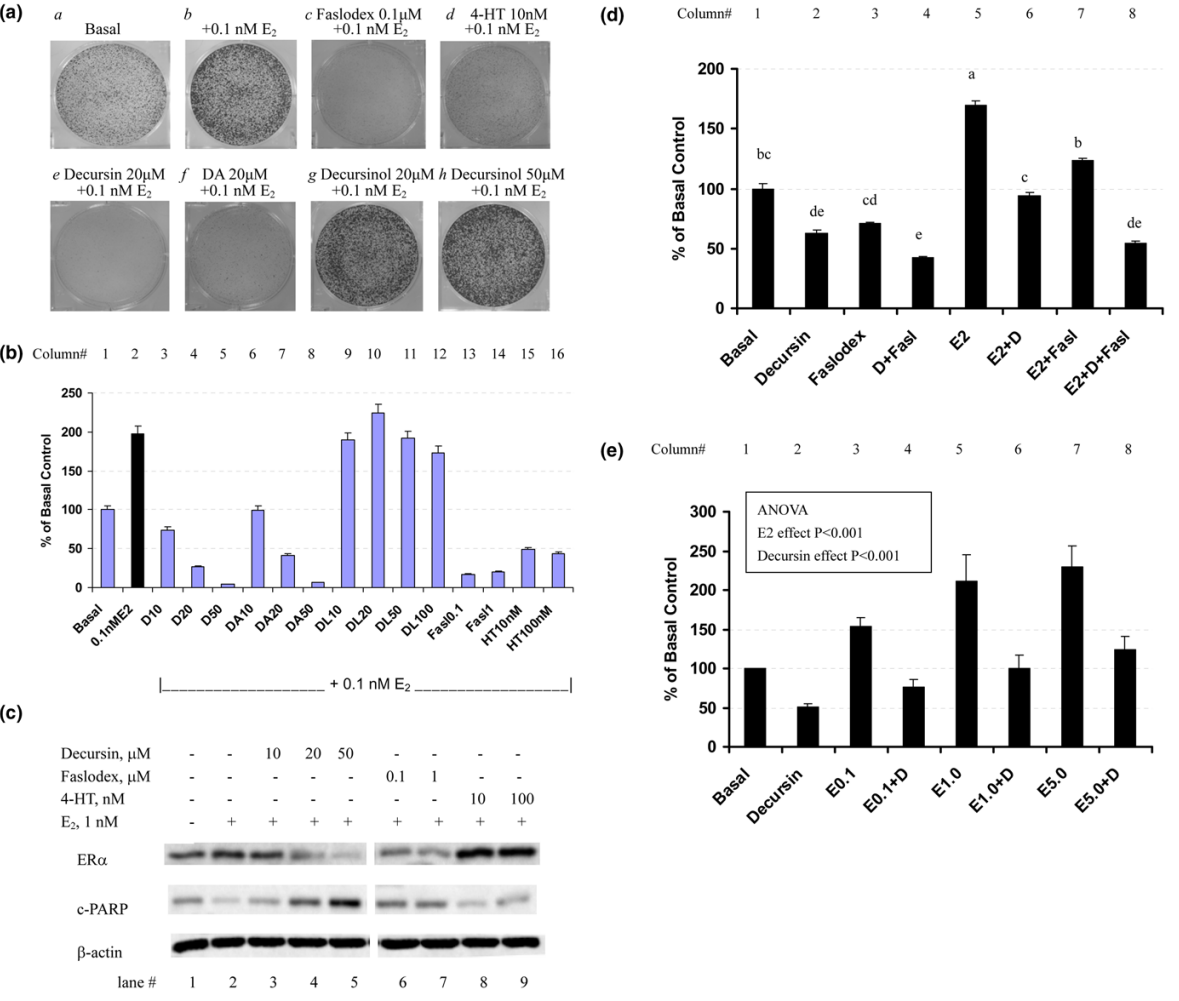
Comparison of pyranocoumarins with antiestrogens Faslodex™ and 4-hydroxytamoxifen. **(a), (b) **Effects of decursin (D) and decursinol angelate (DA) versus decursinol (DL) on estrogen-stimulated growth of MCF-7 cells as sparsely seeded colonies. MCF-7 cells (~10% confluent) were seeded in six-well plates in phenol-red free improved minimum essential medium (PRF-IMEM) with 5% charcoal-stripped serum without insulin with solvent, a pyranocoumarin or a known antiestrogen for 3 days. Without medium change, 0.1 nM 17β-estradiol (E_2_) was added for another 9 days. After fixation with 1% glutaraldehyde, the cells remaining attached were stained *in situ *with 0.02% crystal violet and photographed. (a) Image of representative wells. (b) Cell number estimated by dissolving the crystal violet in 70% ethanol (4 ml/well) and then optical density (OD) values measured at 570 nm with the reference filter of 405 nm. Results (mean ± standard error) represent triplicate wells. Analysis of variance (ANOVA): D at concentrations 10–50 μM (D10-D50) versus (+)E_2_, *P *< 0.0001; DA10-DA50 versus (+E_2_), *P *< 0.0001; DL10-DL100 versus (+)E_2_, *P *> 0.5; Faslodex™ versus (+)E_2_, *P *< 0.0001; 4-hydroxytamoxifen (4-HT) versus (+)E_2_, *P *< 0.001. **(c) **Comparison of effects of D with those of Faslodex™ and 4-HT on ERα and cleaved poly(ADP-ribose) polymerase (c-PARP) in MCF-7 cells in the hormone deprivation/restimulation model. Fresh medium with 1 nM E_2 _without or with D, or with known antiestrogen was replenished to respective flasks for 24 hours and the cellular extract analyzed by western blot. **(d) **Effect of combining D and Faslodex™ on basal and estrogen-stimulated colony growth in PRF-IMEM in six-well plates. Various treatments started at seeding for 5 days. Each bar represents mean ± standard error of the mean for triplicate wells. Results for one of two experiments, with identical patterns of responses. Concentrations: D, 10 μM; Faslodex™, 0.1 nM; estradiol, 0.1 nM. Columns sharing the same letter are not different at *P *= 0.05. **(e) **Effect of D on colony growth of MCF-7 cells stimulated by increasing levels of estradiolin six-well plates. Various treatments started at seeding for 5 days. Each bar represents mean ± standard error of the mean for three independent experiments, each with duplicate or triplicate wells. D concentration, 10 μM. ANOVA: E_2 _effect, *P *< 0.001; D effect, *P *< 0.001; E_2 _× D interaction, *P *> 0.5.

To specifically address the inhibitory activity of decursin or DA on estrogen-stimulated cell growth, we seeded MCF-7 cells in PRF-IMEM with charcoal-stripped FBS for 3 days to decrease estrogen signaling, and then treated them with decursin, DA or decursinol in the presence of 0.1 nM 17β-estradiol for 9 days. Figure 4a shows representative wells from the different treatments. Estrogen at this physiologically relevant concentration stimulated the colony growth approximately twofold (Figure 4b, column 2 versus column 1; *P *< 0.01). The specificity of estrogen signaling was verified by the steroidal pure antiandrogen Faslodex™ [15,30,36-38] (Figure 4a, *c *versus *b *and Figure 4b, columns 13 and 14 versus column 2; *P *< 0.0001) and by the nonsteroidal partial antagonist 4-HT (Figure 4a, *d *versus *b *and Figure 4b, columns 15 and 16 versus column 2; *P *< 0.0001), which effectively inhibited the estrogen-stimulated colony growth. Decursin (Figure 4a, *e*) and DA (Figure 4a, *f*) decreased estrogen-stimulated colony growth of MCF-7 cells with similar potency (Figure 4b, columns 3–5 and 6–9 versus column 2). Approximately 10 μM each of decursin and DA was required to block the stimulated response by 0.1 nM 17β-estradiol (Figure 4b, columns 3 and 6 versus 2). Decursinol, however, lacked any inhibitory activity within the concentration range tested (Figure 4a, *g *and *h *versus *b *and Figure 4b, columns 9–12 versus column 2). Additional experiments indicated even a mild stimulation effect ('agonist') of decursinol in the absence of estrogen (data not shown).

To contrast the actions of decursin with the two well-characterized antiestrogens, we analyzed MCF-7 cells treated with decursin, Faslodex™ or 4-HT after 24 hours of estradiol stimulation for ERα and for cleaved PARP by western blot analysis (Figure 4c). Decursin caused a concentration-dependent decrease of ERα protein with a reciprocal increase of the cleaved PARP (Figure 4c, lanes 4 and 5 versus lane 2). Faslodex™ (Figure 4c, lanes 6 and 7) effectively decreased ERα abundance, and 4-HT (Figure 4c, lanes 8 and 9 versus lane 2) increased the ERα level – both changes as expected [15,37]. These latter two agents caused no increase in the cleavage of PARP.

The ability for decursin and DA to downregulate ERα (Figure 4c) suggested mechanistic similarities with the pure antiestrogen Faslodex™ and distinctions from 4-HT. Indeed, results presented in Figure 4d show that decursin and Faslodex™ suppressed MCF-7 cell colony growth in an additive manner in the absence (Figure 4d, columns 1–4) or in the presence (Figure 4d, columns 5–8) of estradiol.

Since these results were consistent with an inhibition of estrogen-stimulated mitogenic signaling by decursin and DA, but not by decursinol, we tested whether greater levels of estradiol could overcome the inhibitory effects of decursin. Sparsely-seeded MCF-7 cells in the absence or presence of 10 μM decursin were treated with solvent vehicle or were stimulated with 0.1 nM, 1 nM and 5 nM 17β-estradiol for 5 days. Estrogen stimulation in the absence of decursin increased the colony growth significantly in a concentration-dependent manner (Figure 4e, columns 3, 5, and 7 versus column 1). Regardless of the strength of estrogen stimulation (0.1–5 nM, a 50-fold range), decursin inhibited the growth of MCF-7 cells (Figure 4e), producing a consistent relative inhibitory effect when normalized to the respective estrogen dosages. These results suggest that the action of decursin was probably not accounted for as an antagonist competitor for estrogen ligand binding to ERα.

### Decursin and decursinol angelate suppressed ERα protein abundance, mRNA level and estrogen early signaling in MCF-7 cells

**Figure 5:**
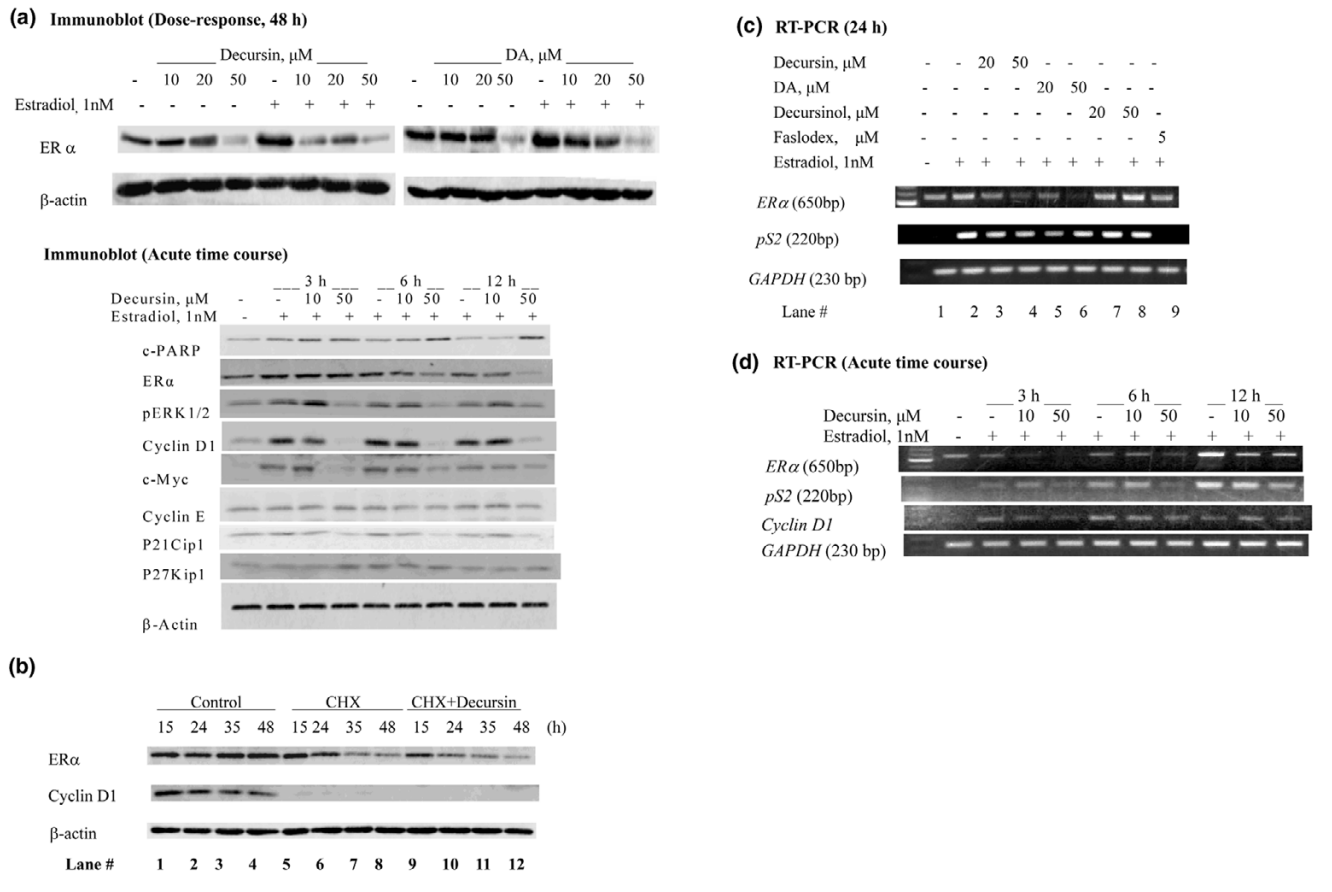
Effects of pyranocoumarins on estrogen receptor alpha protein stability and mRNA level in MCF-7 cells. **(a) **Effect of decursin (D) and decursinol angelate (DA) on estrogen receptor (ER)α protein abundance in the absence and presence of estrogen. MCF-7 cells were cultured in phenol-red free improved minimum essential medium (PRF-IMEM) with 5% charcoal-stripped FBS (CSS) and without insulin for estrogen starvation for 3 days. For estrogen stimulation, D or DA was added 2 hours before 1 nM 17β-estradiol (E_2_) was added without medium change. Cell lysates were prepared 48 hours after D or DA treatment for western blot analyses. In the time course, D and E_2 _treatments initiated simultaneously. c-PARP, poly(ADP-ribose) polymerase. **(b) **Effects of D (50 μM) on ERα protein stability in MCF-7 cells treated in complete medium with cycloheximide (CHX, 50 μg/ml) for 15, 24, 35, and 48 hours. Cell lysates analyzed by western blot for ERα and the short-lived cyclin D_1 _to establish potency of CHX. **(c) **Effects of D, DA and decursinol (DL) on the steady-state level of mRNA transcripts of *ERα *and the estrogen-responsive gene *pS2 *detected by RT-PCR. MCF-7 cells were cultured in PRF-IMEM with 5% CSS and without insulin for estrogen starvation as above. D, DA, DL or Faslodex™ was added 2 hours before 1 nM E_2 _without medium change. After 24 hours, cells were harvested for RNA isolation and RT-PCR. The housekeeping glyceraldehyde-3-phosphate dehydrogenase (*GAPDH*) gene serves as a loading control. **(d) **Acute time-course effect of decursin on *ERα *and estrogen-responsive genes *pS2 *and *cyclin D*_1 _detected by RT-PCR. D and E_2 _treatments initiated simultaneously.

We subsequently focused on elucidating the mechanisms by which the effective compounds affected ERα abundance and signaling. Decursin and DA suppressed the ERα protein level in MCF-7 cells in the absence or presence of estrogen stimulation when treated for 48 hours in PRF-IMEM with charcoal-stripped serum (Figure 5a).

An acute time-course experiment indicated that the downregulation of ERα occurred as early as after 6 hours of exposure to decursin at a dose (50 μM) that increased PARP cleavage (Figure 5a). This dose of decursin abolished the phosphorylative activation of ERK1/2 by estradiol, and abolished the upregulation of the products of early estrogen-stimulated genes such as cyclin D_1 _(early G_1 _phase) and c-Myc. The S-phase-specific cyclin E was not affected, nor were the expression levels of P21Cip1 and P27Kip1 increased during this timeframe by decursin.

To examine whether decursin increased ERα protein degradation, we blocked new protein synthesis in MCF-7 cells in complete medium with cycloheximide (50 μg/ml) and analyzed the ERα protein abundance by western blot analysis (Figure 5b). The short-lived cyclin D_1 _was decreased by cycloheximide to below the detection level (Figure 5b, lanes 5–8), affirming the effectiveness of cycloheximide to block protein synthesis. The level of ERα protein at 15 and 24 hours of cycloheximide treatment (figure 5b, lanes 5 and 6) was further decreased by decursin (figure 5b, lanes 9 and 10). The data were consistent with an enhanced degradation of ERα protein in decursin-treated MCF-7 cells.

To determine whether decursin and DA affected the ERα mRNA transcript abundance and to examine their impact on transactivation of ERα-target genes, we examined the steady-state mRNA level of *ERα *and the known estrogen-regulated gene *pS2 *[39] using RT-PCR after cells had been exposed to pyranocoumarin compounds for 24 hours (Figure 5c). As expected, estrogen stimulation (Figure 5c, lane 2 versus lane 1) greatly increased the *pS2 *mRNA, and Faslodex™ (Figure 5c, lane 9 versus lane 2) blocked this estrogen-stimulated change. Decursin or DA reduced the *ERα *mRNA abundance in a concentration-dependent manner to below the basal level (Figure 5c, lanes 3 and 4, lanes 5 and 6 versus lane 2). In contrast to the dramatic impact of decursin or DA on the *ERα *mRNA level, the *pS2 *mRNA level was only modestly decreased by decursin or DA. The *ERα *and *pS2 *mRNA levels were not decreased by decursinol treatment (Figure 5c, lanes 7 and 8 versus lane 2).

In the time-course study (Figure 5d), the *ERα *mRNA level was decreased by decursin (50 μM) exposure as early as 3 hours, and the effect persisted through 12 hours. A suppressing effect of this dose of decursin on the *pS2 *mRNA level was detectable by 6 hours. Interestingly, the estrogen-stimulated induction of the cyclin D_1 _mRNA level was decreased by decursin only at the 3 hour timepoint. Combined, these data suggest that decursin can downregulate ERα expression at the transcriptional level as well as by post-translational degradation.

### Decursin increased ERα cytosol retention and inhibited transactivation of estrogen-response element

**Figure 6:**
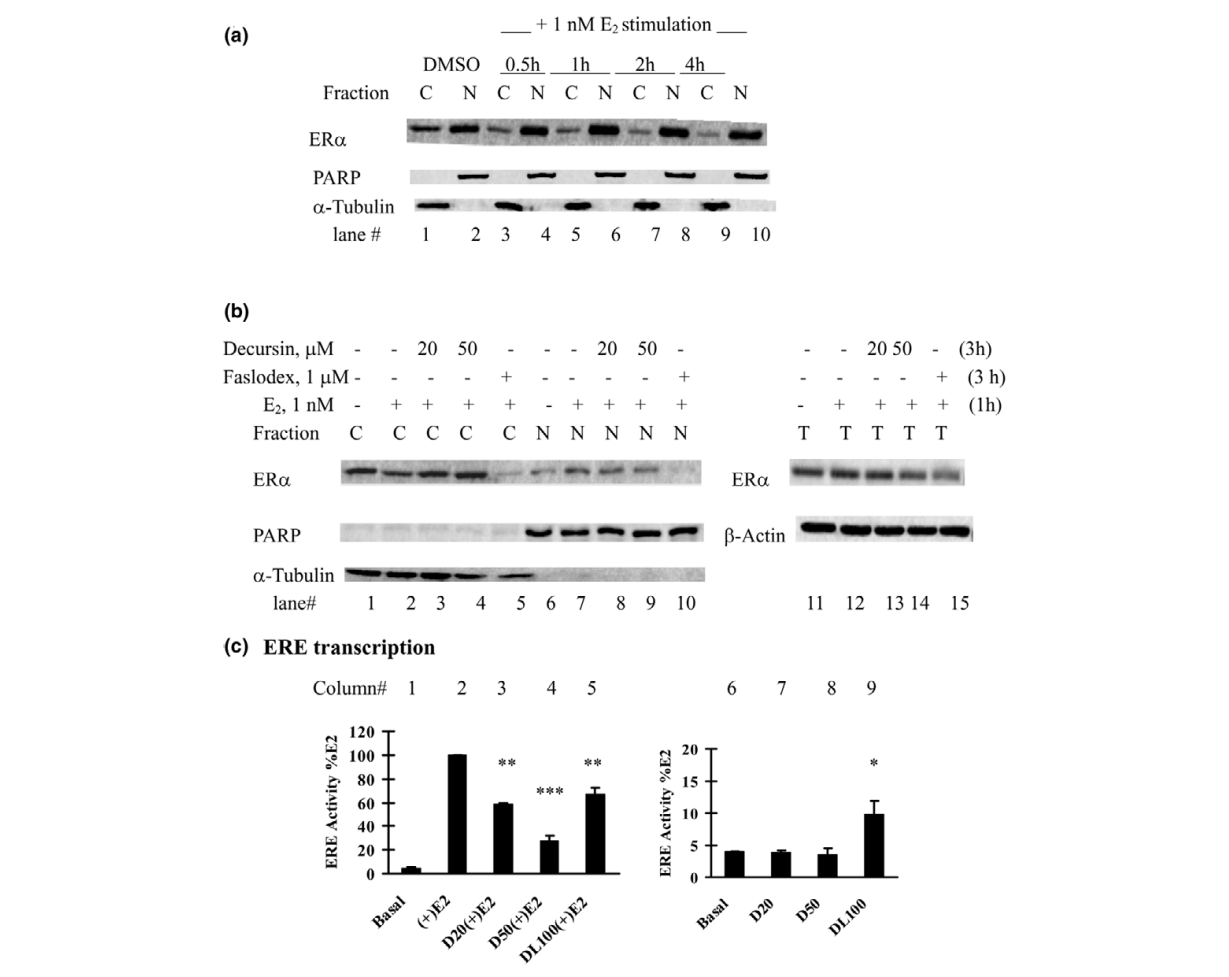
Effects of pyranocoumarins on estrogen receptor alpha nuclear translocation and transactivation in MCF-7 cells. **(a), (b) **Inhibitory effects of decursin on estrogen receptor (ER)α nuclear translocation in MCF-7 cells. (a) The time course of ERα nuclear translocation in MCF-7 cells stimulated by 17β-estradiol (E_2_) was first established in estrogen-starved MCF-7 cells. Fresh medium without or with 1 nM E_2 _was added. At different time points, nuclear (N) and cytosolic (C) fractions were prepared for western blot analyses. Poly(ADP-ribose) polymerase (PARP) and α-tubulin were detected as markers of N and C proteins, respectively. DMSO, dimethylsulfoxide. (b) For the decursin experiment, estrogen-deprived MCF-7 cells were pretreated with decursin for 2 hours and were stimulated with 1 nM E_2 _for 1 hour (total decursin exposure time, 3 hours). N and C fractions were prepared for western blot analyses. One-tenth nuclear fraction was used compared with the time-course experiment to show the difference. The total lysate ERα level was determined (right panel). **(c) **Differential effects of decursin versus decursinol on estrogen-response element (ERE) activity in MCF-7 cells: left, with E_2_; right, without, E_2_. An aliquot of 1 × 10^5 ^MCF-7 cells was placed in a 12-well plate and cotransfected with ERE-luciferase reporter plasmid and pSV-β-galactosidase reporter in phenol-red free improved minimum essential medium (PRF-IMEM) without serum and insulin. After transfection for 24 hours, the cells were treated with different concentrations of decursin (D, 20 μM, 50 μM), decursinol (DL, 100 μM) or DMSO in the absence or presence of 1 nM E_2 _for 24 hours. Cells extracts were prepared for luciferase activity and β-galactosidase activity. Mean ± standard error, three independent experiments. *P < 0.05, **P < 0.01, ***P < 0.001 versus basal or E_2_-stimulated control.

Because decursin and DA potently inhibit the cytosol to nuclear translocation of AR upon androgen stimulation in prostate cancer cells [26,27], we investigated whether a similar inhibitory effect was exerted on ERα. We first determined the optimal timeframe to study ERα nuclear translocation in MCF-7 cells. As shown in Figure 6a (lane 2 versus lane 1), the ERα was predominantly localized in the nucleus in MCF-7 cells grown in PRF-IMEM medium without serum and insulin for 48 hours. This was in contrast to the predominant cytosolic distribution of AR in androgen-deprived prostate cancer cells [26]. Nevertheless, a decrease of cytosolic ERα (Figure 6a, lane 3 versus lane 1) and a reciprocal increase of nuclear ERα (Figure 6a, lane 4 versus lane 2) could be detected as soon as 30 minutes of stimulation by 17β-estradiol, and the changes persisted through 4 hours of stimulation (Figure 4a).

For experiments with decursin or Faslodex™, we pretreated estrogen-deprived MCF-7 cells for 2 hours with these agents and then stimulated with 1 nM 17β-estradiol for 1 hour (total decursin exposure time of 3 hours). We used one-tenth of the nuclear extract of that used in the time-course experiment to increase the sensitivity to detect a difference in the nuclear ERα abundance. The results showed that decursin prevented the cytosolic decrease of ERα induced by 17β-estradiol (Figure 6b, lanes 3 and 4 versus lane 2), and dampened the increase in nuclear ERα in a reciprocal manner (Figure 6b, lanes 8 and 9 versus lane 7). In contrast, Faslodex™, as expected [15], induced a sharp decrease of the cytosolic ERα as well as the nuclear ERα (Figure 6b, lanes 5 and 10). The reduction of ERα induced by Faslodex™ was reflected in the total cell lysate (Figure 6b, lane 15 versus lanes 11 and 12).

To further evaluate the effect of decursin on ERα transactivation of estrogen-stimulated genes, we used a transient transfection assay with luciferase reporter plasmid driven by multiple copies of the ERE [32]. The reporter assay showed a dramatic activation of ERE-promoter activity by 17β-estradiol (~25-fold) (Figure 6c, column 2 versus column 1). Decursin caused a concentration-dependent decrease of estrogen-stimulated ERE transactivation (Figure 6c, columns 3 and 4 versus column 2). Decursinol, in contrast, exerted a moderate inhibition of the estrogen-stimulated transcription (Figure 6c, column 5). In the absence of 17β-estradiol, however, decursinol had a very mild stimulation effect on basal ERE activity (Figure 6c, column 9 versus column 6) whereas decursin lacked such an 'agonist' effect (Figure 6c, columns 7 and 8 versus column 6).

## Discussion

In the present study we examined the growth inhibitory effects of decursin and DA on two human breast cancer cell lines, and the probable mechanisms, to provide a scientific rationale to support the potential utility of these compounds and/or their derivatives as breast cancer chemopreventive agents. A number of significant and novel findings in the present study are worthy of further comment.

The first observation is the structural basis for the *in vitro *breast cancer growth inhibitory effects of decursin and its natural analogs. We found that decursin and DA, structural isomers on the side chain (Figure 1a), exerted comparable potency in terms of cell-cycle arrest (Figures 2a and 3a), apoptosis induction (Figures 2b,c and 3b,c) and impact on selected molecular targets, such as inactivation of ERK1/2 (Figures 2c and 3c), in both MCF-7 cells and MDA-MB231 cells. Decursinol, without the side chain, lacked these effects at comparable concentrations. These results are consistent with the structure-activity patterns reported for decursin and DA to inhibit androgen/AR signaling and to induce cell-cycle arrest and apoptosis in prostate cancer cells [27]. These studies together support the critical requirement of the side chain attached to the pyranocoumarin core structure to exert these diverse actions related to steroid hormone signaling as well as cell-cycle arrest and proapoptotic pathways.

Second, the inhibitory actions reported here for decursin and DA against estrogen-induced signaling events and cell proliferation in the MCF-7 cell model highlight potential specificity for these compounds to attenuate breast cancer risk in premenopausal women. In addition, the observed cell-cycle arrest and apoptotic effects of these two compounds on MDA-MB231 cells suggest that these agents may also be useful for the control of estrogen-independent breast cancer growth. In MCF-7 cells, the agents' effects on the cell cycle and apoptosis were associated with decreased ERα abundance; in MDA-MB231 cells, their effects on cell-cycle arrest and apoptosis were closely accompanied by an induction of ERβ and a paradoxical loss of P21Cip1 expression. Decursinol exerted minimal effect on these molecules. Although both receptors are capable of regulating gene transcription through binding the ERE, ERα and ERβ have been shown to be differentially activated by various ligands [7,8] – whereas ERα is able to regulate endogenous genes in a ligand-dependent manner, ectopically expressed ERβ has been shown to inhibit MDA MB-231 cell proliferation in response to added estrogen [8]. A study has shown that the forced expression of ERβ in breast cancer cells inhibits their growth *in vitro *through an induction of G_2 _arrest [34]. A recent paper showed that an inducible expression of ERβ through a Tet-Off system *in vivo *potently inhibited the growth of T47D xenograft and angiogenesis [40]. Whether ERβ induction by these compounds is restricted to estrogen-independent breast cancer cells and the significance of this induction in growth arrest induced by decursin or DA await further analyses.

Third, comparison of the actions of decursin and DA with those of Faslodex™ and 4-HT in MCF-7 cells revealed both similar and distinct mechanisms from these well-characterized antiestrogens to inhibit estrogen-stimulated growth (Figures 4c and 5c). Tamoxifen is a triphenylethylene antiestrogen with partial ER agonist activity and has been used for a couple of decades for the classic hormonal treatment of breast cancers expressing ERs [14,15]. In spite of its partial agonist activity, tamoxifen has been approved for use as chemopreventive agent for breast cancer in high-risk women [14]. On the other hand, ICI 182,780 (now called fulvestrant or Faslodex™) is the only steroidal antiestrogen that has reached clinical development. Both compounds are competitive inhibitors for the binding of 17β-estradiol to ERα, but their mechanisms of action are quite different. Tamoxifen inhibits the activation function 2 of ERα-mediated transactivation, probably via the recruitment of corepressors. Yet this type of antagonist does not interfere with the activation function 1-mediated transactivation of ERα. Tamoxifen and its active metabolite 4-HT have also been known to cause ER nuclear accumulation. By contrast, Faslodex™ suppresses both activation function 1 and activation function 2 ERα transactivation functions, and prevents nuclear transport of the receptor. In addition, Faslodex™ reduces the half-life of ER protein, leading to receptor content downregulation [15,37].

Decursin shares some mechanisms of actions with Faslodex™, including a downregulation of the ERα protein level (Figures 4c and 5a) through increased protein degradation (Figure 5b) and decreased abundance of ERα mRNA (Figure 5c). Furthermore, decursin (Figures 4d,e and 6c) and DA (data not shown) did not exert any 'agonist' activity in the absence of estrogen. Such a property is desirable for breast cancer prevention in contrast to tamoxifen, which exerts a partial agonist activity, and its long-term use increases the risk of endometrial cancer [10,11,14]. At the molecular target level, decursin differed from the active tamoxifen metabolite 4-HT, which increased ERα protein abundance (Figure 4c) as has been well documented [14,15].

Fourth, the present study extended our understanding of the inhibitory actions of decursin and DA on sex steroid receptor signaling from prostate cancer cells to breast cancer cells. Similarities include observations that these agents inhibit the ligand-induced nuclear translocation of ERα (Figure 6b) and AR [26,27], induce an increased degradation of ERα (Figure 5b) as well as AR [27], and attenuate their transactivation of respective promoter sequences (Figure 6c) [27]. In contrast to the lack of any impact by decursin or DA on the *AR *mRNA level in LNCaP cells [26,27], however, a sharp and rapid downregulation of *ERα *mRNA by these compounds was observed in MCF-7 cells (Figure 5c), suggesting different pathways were targeted in organ-specific ways.

## Conclusion

In summary, our data provide support for decursin and DA as potential novel breast cancer chemopreventive agents by targeting ERα signaling through inhibiting its transcription, stability and abundance as well as nuclear translocation in estrogen-dependent MCF-7 cells, and by inducing ERβ in estrogen-independent MDA-MB231 cells, leading to cell-cycle arrest and proapoptotic actions. In both cell lines, decreased ERK1/2 phosphorylation was associated with these cellular effects, whereas the effects of these compounds on cell-cycle regulatory proteins cyclin D_1_, P21CIP1 and P27KIP1 appeared cell line specific. Our data suggest that these agents may also be used in combination with existing antiestrogen drugs such as Faslodex™ and tamoxifen to increase preventive efficacy and to decrease unwanted side effects. *In vivo *validation of the agents' breast cancer preventive and treatment efficacy in preclinical animal models is warranted.

## Abbreviations

AR = androgen receptor; bp = base pairs; ELISA = enzyme-linked immunosorbent assay; ER = estrogen receptor; ERE = estrogen-response element; FBS = fetal bovine serum; 4-HT = 4-hydroxytamoxifen; IMEM = improved minimal essential medium; PARP = poly(ADP-ribose) polymerase; PCR = polymerase chain reaction; PRF = phenol-red free; RT = reverse transcriptase.

## Competing interests

The authors declare that they have no competing interests.

## Authors' contributions

CJ, JG, ZW, S-HK and JL designed the experiments, analyzed and interpreted the data, and drafted the manuscript. CJ, JG, ZW and BX carried out the cell culture experiments, cell growth and apoptosis determinations, western blot analyses and RT-PCR analyses. H-JL, E-OL and S-HK prepared the pyranocoumarin compounds. All authors read and approved the final manuscript.
